# The Risk and The Course of Cancer Among People with Severe Mental Illness

**DOI:** 10.2174/17450179-v17-e211208-2021-HT2-1910-8

**Published:** 2023-12-03

**Authors:** Luigi Grassi, Daniel McFarland, Michelle Riba

**Affiliations:** 1 Department of Neuroscience and Rehabilitation, DInstitute of Psychiatry, University of Ferrara and University Hospital Psychiatric Unit, Ferrara, Italy; 2 Department of Medicine, Hofstra University, Northwell Health, Lenox Hill Hospital, New York, NY, US; 3 Department of Psychiatry, University of Michigan, Ann Arbor, MI, USA; 4 Psycho-oncology Program, University of Michigan Depression Center and Rogel Cancer Center, Ann Arbor, MI, USA

**Keywords:** Cancer, Incidence, Course, Schizophrenia, Bipolar disorders, Depression

## Abstract

The paucity of data regarding patients with Serious Mental Illness (SMI) and cancer is alarming given the fact that people with SMI, especially schizophrenia, bipolar disorders and severe depressive disorders, have in general poorer access to physical health care and higher morbidity and mortality because of physical illnesses. The aims of this review were to examine the current evidence from existing literature on the risk of developing cancer and its course among people with SMI. Equivocal results emerge regarding the risk of developing some kind of cancer among people with SMI, with contrasting data on a possible higher, similar or lower risk in comparison with the general population. In contrast, a series of studies have pointed out that patients with SMI who develop cancer are less likely to receive standard levels of cancer care, both in terms of screening, diagnosis and treatment. Also, the mortality for cancer has been confirmed to be higher than the general population. A global sensitization about these problems is mandatory in an era in which community psychiatry has been developed in all countries and that policies of prevention, treatment, follow up, and palliative care should regard all the segments of the population, including people with SMI, through an interdisciplinary approach.

## 1 INTRODUCTION

The paucity of data regarding cancer in people with Serious Mental Illness (SMI) is alarming and should be the focus of attention. The World Health Organization (WHO) estimates that by 2030 cancer incidence will increase by 40% in high-income countries and more than 80% in low-income countries (WHO, 2018) [1]. In 2018, there were 18.1 million new cancer cases and 9.6 million cancer deaths, which will escalate to over 13 million deaths by 2030 [2]. These data are even more warning if we consider that people with SMI, namely schizophrenia, bipolar disorders and severe depressive disorders, have in general poorer access to physical health care [3], an increased incidence of physical diseases (*e.g*. diabetes, heart disease), and a life expectancy of 15-20 years shorter with respect to the general population [4-8].

Regarding cancer, it has been demonstrated that people with SMI are less likely to receive standard levels of cancer care and that they have higher mortality from cancer than the general population [9]. A global sensitization about the need for patients with SMI to have proper access to cancer care is therefore extremely important in an era in which community psychiatry has developed in all countries and that the recommended WHO health policies underscore the mandatory need and commitment for prevention and screening, treatment and follow up for physical care among people with SMI [10].

Therefore, given the urgent need to increase the knowledge in this area, this paper reviews the current evidence from existing literature on the risk of developing cancer and the course of the disease among people with SMI, specifically schizophrenia / schizophrenic spectrum disorders and mood (bipolar, major depression) disorders.

## 2 METHODOLOGY

We conducted a literature review on the risk of developing cancer among patients with SMI and the course of the disease cancer in terms of mortality. We used the following search keywords: “cancer” “AND/OR” “schizophrenia”, “bipolar disorders” “major depression” and “mortality”. According to the needs, the keywords were searched in the publication title or abstract through Pubmed/Medline, EMBASE, PsycLit, PsycInfo, Cochrane Library, ISI Web of Knowledge as databases. From the references found in the electronic databases, a manual search of these references was also performed. The following inclusion criteria were established: papers in English language, published in the last thirty years (from 1990 to 2020), including metanalyses and reviews, in peer-reviewed journals. Exclusion criteria were: communications at meetings or abstracts from conference proceedings. After reading the full-text, the papers were analyzed with respect to their content, and those with content were not fully within the scope of this narrative review were eliminated.

## 3 RESULTS

### 3.1 Risk of Cancer Among People with SMI

The risk of developing cancer among people with SMI has been studied with different results according to the methodology of epidemiological studies as well as the type of mental disorder considered in the studies, namely schizophrenia and related disorders, and mood disorders, including depression and bipolar illness.

#### 3.1.1 Schizophrenia Spectrum Disorders

The literature regarding the risk of developing cancer in people with schizophrenia has been the subject of a number of studies since the ‘70s. The psychosomatic hypothesis (accor-ding to the psychoanalytically-based psychophysiological model) that cancer could have been considered the expression of a maximal regression at a somatic level of what psychotic illness is at a mental level, (with therefore psychosis being a protective factor for cancer and vice versa) [11] was never clearly confirmed by evidence-based data. Over the last 30 years, some epidemiological studies have revealed a lower risk of certain types of cancer (*e.g*. breast, prostate, lung, and gastrointestinal cancer) in patients affected by schizophrenia in comparison with the general population [12]. In a nine-year follow-up period, Chou *et al*. [13] found that patients with schizophrenia had a significantly lower cancer incidence than those in the control group (1.9% incidence vs 2.9%) in both males and females. Other studies, however, have indicated a similar incidence of cancer among patients with SMI and the general population [14], whereas an increased risk for cancer was found among people with schizophrenia living in Asia–Africa, in contrast with people born in Europe–America, in whom the overall risk was lower [15]. On these figures, it has been underlined the need to carry out multinational studies which should include identification of cancer-related risk factors among patients with schizophrenia. Also, the site of cancer has been reported as important, with some data indicating a standardized incidence ratio (SIR) of lung cancer marginally reduced (SIR=0.86) for male patients and an increased risk for breast cancer in female patients with schizophrenia (SIR=1.20) [16]. In a 25-year Northern European follow-up study, Petterson *et al*. [17] found a higher risk for breast, lung, esophageal and pancreatic cancer and a lower risk of prostate cancer, confirming previous studies [18].

When meta-analyses were carried out, the contradictory findings that emerged left this epidemiological field not clear and the problem remained unsolved [19, 20]. In a systematic review of 13 studies involving data from over 6,000 female patients with schizophrenia fin comparison to age matched general populations (time span: 1986 to 2008), Bushe *et al*. [21] reported contradictory results, with 52% increase in risk to 40% decrease, and six of 13 studies showing an increased or marginally increased incidence of breast cancer. The meta-analysis by Catts *et al*. [22] including 16 studies of patients with schizophrenia showed that the general incidence of cancer was not increased (SIR=1.05), although breast cancer incidence was higher among female patients. In a more recent meta-analysis of 12 cohort studies including 125,760 women, schizophrenia was associated with an increased breast cancer incidence compared with the general population [23]. Another meta-analysis [24] of 16 studies (480,356 patients with schizophrenia and 41,999 cases of cancer) showed that the prevalence of prostate and colorectal cancer was lower in male patients with schizophrenia, while the prevalence of lung cancer in female patients was higher. In contrast, a further recent metanalysis of 12 studies for a total of 496,265 patients with schizophrenia, showed no difference in lung cancer incidence [25].

#### 3.1.2 Mood Disorders

With respect to mood disorders, the data regarding the incidence of cancer are also not uniform. [26] Regarding depression, a series of follow-up epidemiological studies seemed not to indicate an increased risk for developing cancer among patients affected by depressive clinical conditions. This was evident in the GAZEL study in France, where a target population of 20,625 French national gas and electricity company employees were followed-up for years [27]. Similar data were reported in other studies, namely: a population-based cohort of 6,848 persons free of cancer who were followed from 1965 to 1982 as part of the Alameda County study in the USA [28]; the US National Health and Nutrition Examination Survey I Epidemiologic Follow-up Study [29]; the USA multi-site epidemiologic survey of community-dwelling adults Epidemiologic Catchment Area (ECA) Program, in East Baltimore [30]; the UK Whitehall II study involving participants of office staff with an age from 33 to 55 years working in civil service departments in London [31], as well as in other studies [32-34].

However there are data running in the opposite direction and showing, among people with depression recruited from large-based population studies, either a higher risk for some kind of cancers such as hormonally-related cancers (*e.g*. breast, prostate) [35, 36], lung cancer possibly related to smoking habits [37], and colon- cancer in women [38] or a higher risk both in old people [39] and in all ages [40]. In a recent large 19-year prospective cohort study in Korea (Korean Cancer Prevention Study KCPS) involving 601,775 people with an age ranging from 30 to 64 years, Chang *et al*. [41] it was shown for example that, after adjusting for possible confounders, men affected by minor depression had a higher risk of prostate cancer (HR 1.13), while in women cervical cancer was inversely associated with major depression (HR 0.90). In general, major depression was positively associated with overall cancer incidence in men, while the opposite was found in women. In contrast, in a Taiwanese follow-up study of patients with major depression, Chen *et al*. [42], found a higher risk of developing cancer.

Once again, meta-analyses on this topic did not help to clarify the problem. In a review of the most relevant studies of breast cancer patients, there was in fact, a trend suggesting that depressed patients were more likely to develop cancer [43, 44]. A further metanalysis of 9 studies confirmed that patients with depressive disorder had an increased risk for cancer (OR=1.26); however, this effect was observed only in low-quality studies (OR=1.31) and not in high-quality studies (OR=1.15) [45]. More recently, a large metanalysis of all the published studies on this topic, indicated that overall, both depression and anxiety were indeed associated with an increased incidence of cancer (adjusted RR= 1.13) with a higher risk in some sites of cancer (*i.e*. lung, oral cavity, prostate and skin). Also a higher cancer-specific mortality risk was shown for some cancers (*i.e*. lung, bladder, breast, colorectum, hematopoietic system, kidney and prostate) [46].

As far as bipolar disorders are concerned, an Israeli study indicated that both men and women with bipolar disorder (SIR=1.59 and =1.75, respectively) had a higher risk for cancer [47]. A large Swedish study [48] of 15,386 patients with bipolar disorders and 39,182 patients with major depression found a marginally higher risk of cancer in both populations. In a large Taiwanese follow-up study (from 1997 to 2009) which involved a total of 71,317 patients with schizophrenia and 20,567 patients with bipolar disorder, Lin *et al*. [49] found a higher incidence of all types of cancer in both samples, although the higher risk was found in males but not females. Likewise, by comparing the incidence of cancer amongst 3,317 patients with schizophrenia or bipolar disorder in a ten-year follow-up period (1994-2004) the risk of cancer was 2.6 times higher in both cohorts in comparison with the U.S. population [50]. A higher risk for breast cancer was also found among Swedish female patients with bipolar disorder [51] and in a study of patients with mood disorders in Taiwan. In the latter, however, the risk of cancer [mainly tobacco- and/or alcohol-related cancers] was higher in patients with unipolar depression (9,826 subjects, SIR=2.01) than bipolar disorders (10,207 subjects SIR=1.39) [52]. In contrast, in a large study of 3,357 subjects with bipolar I disorder, Carney *et al*. [53] found a higher risk for cancer.

Some data are available in patients with bipolar disorder compared to patients with schizophrenia. Hippisley-Cox *et al*. [54] showed that incidence of colon cancer was lower among people with bipolar disorders or the general population with respect to patients with schizophrenia. Also, no significant increase in the risk for cancer was found among patients with schizoaffective disorders [55] and among a large population of 20,632 patients with several SMI (schizophrenia, schizoaffective disorders, and bipolar disorders) in comparison with 116,152 people without SMI within the UK Health Improvement Network (THIN) study [56].

### 3.2 The Course of Cancer

While the incidence of cancer in patients with SMI is equivocal in some cancer groups or lower than what might be expected given lifestyle habits (*e.g*., smoking, diet), patients with SMI who develop cancer unequivocally have worse prognosis than patients without SMI.

Disparities in health services provided to people with SMI who developed cancer are consistently documented. For example, it has been shown that psychiatric patients were more likely to be diagnosed with metastatic cancer and to receive lower quality specialized interventions, with consequent increased mortality [57]. Likewise, in a study of 16,636 elderly women, patients with comorbid anxiety and depression were more likely to receive a late diagnosis (≥90 days from symptom recognition), while those with SMI were more likely to not receive early treatment, with a delay ≥60 days from diagnosis [58]. In a study of 49,985 patients with locoregional high-grade prostate cancer, Fried *et al*. [59] showed that patients with SMI (*i.e*. bipolar disorder, schizophrenia, other psychotic disorders) were less likely to receive proper treatment (*i.e*. surgery, radiation and hormone therapy) in the year after cancer diagnosis. In a Danish study involving 56,152 women with early-stage breast cancer diagnosed in 1995-2011, patients with schizophrenia spectrum disorders (n=409) were less likely to receive cancer treatment according to proper guidelines [60]. This was a possible factor related to worse survival in the patient group. Similar data were reported in a further Danish study involving 6,068 women treated during their life for clinical depression and who later developed breast cancer. They were less likely to receive proper guideline treatment and this was probably associated with poorer survival [61]. In another study, oral cancer patients with SMI were less likely to receive proper treatment (*i.e*. surgery with or without adjuvant therapy) and had a 1.58-times risk of death in comparison with patients without SMI [62].

Again, in a study carried out in New Zealand mortality for breast and colorectal cancer in a 5 year-period was examined. Among 8,762 people with breast and 4,022 people with colorectal cancer, 440 and 190 (breast and colon-cancer, respectively) had contacts with psychiatric services for schizophrenia, schizoaffective or bipolar disorders. It was shown that people with SMI received diagnosis at a later stage and that contributed to a two and half times higher mortality for breast cancer (after adjusting for several factors) and three times higher mortality for colon cancer than people without SMI, explaining at least a third of the survival difference for this group [63]. In a different study of a large population of 28,477 cancer cases, it was confirmed that people with SMI, depression, dementia and substance use disorders had significantly worse survival after cancer diagnosis independently of cancer stage at diagnosis and other potential confounders [64].

Internalized and externalized stigma are likely to play the biggest role in SMI-related cancer inequities related to the delivery of cancer care [65, 66]. It is known that stigma is one of the most significant factors negatively influencing the chances for psychiatric patients to participate in cancer prevention programs, to receive early diagnosis of cancer, and to have the disease managed properly, with poorer stage of the illness. In a very large meta-analysis of 47 publications involving 501,559 subjects with SMI and more than 4 million as controls, screening for any type of cancer (namely breast, cervical cancer and prostate cancer) was lower among people with SMI [67]. These disparities should be at the center of the attention of health care institutions and stakeholders [68].

It is clear that patient’s inappropriate affect, positive or negative psychotic symptoms, poorer cognitive functioning, disorganized behavior and poorer health behavior (*e.g*. smoking, substance use and/or abuse, poor adherence, social withdrawal) may favour this problem. It has been demonstrated that minimization of physical symptoms, poor cooperation with caregivers or avoidance of the health care system is in fact associated with a lower screening rate [69] and a delay in diagnosis, with presentation at cancer centers in advanced stages of the disease [70, 71]. On the other side, prejudice on the un-treatability of psychiatric disorders, aggressivity and violence of people with SMI, fragmentation of health care services (*e.g*. poor liaison between oncology and mental health services), and the poor vision in person-centered medicine are also part of the problem [72, 73].

## 4 DISCUSSION

In this paper, we have discussed the cancer risk and the trajectory of illness for patients with SMI who are diagnosed with cancer.

As a first general finding, it is still not altogether clear whether patients with SMI have higher cancer incidence than the general population and for which cancer types. There are contradictory results among several epidemiological studies indicating both higher and lower prevalence. Since cancer is a series of many different diseases, a number of factors can determine this inconsistency of data such as cohort characteristics, size and length of follow up, methodological issues, low statistical power, age range of cohorts studied, difficulty in analyzing the many variables intervening in cancer risk (*e.g*., genetic, and so on). As indicated by several authors [74-77], it is in fact quite difficult to control these variables, such as familial or genetic predisposing factors, the endocrine effects of antipsychotic medications, environment in which the person lives, particularly historical and health-service contexts, as well as the effect of missing cancer diagnoses in the population examined. Also, controlling for age and sex in incidence studies would be important since most cancers are diagnosed in patients older than 60 years and the cancers affecting men and women differ, while SMI is more common in young adults [78].

A second general finding is that the course of cancer is worse for patients with SMI. All studies have in fact, demonstrated that cancer-specific prognosis is worse and mortality of cancer is higher both in patients with schizophrenia and bipolar disorders. These data suggest a significant health-related disparity. Data reveal lower participation in screening programs, late diagnosis, and poorer management in patients with SMI compared with that of the general cancer population. These data are consistent with many other studies documenting that patients with SMI have poorer physical health in general and higher mortality caused by all physical illnesses, including cardiovascular diseases, diabetes and other disorders. Based on these important conclusions, the WHO Comprehensive Mental Health Action Plan has repeatedly indicated that Member States and organizations need to develop and implement effective policies, strategies and plans to improve the health, both physical and mental, for people living with SMI [79, 80]. Therefore, reducing the stigma of SMI and promoting healthy behavior and emphasizing healthy lifestyles in vulnerable populations is mandatory. Also, increasing the cancer screening rate, enhancing early detection and treatment for patients with SMI are necessary and mandatory steps in oncology [81, 82].

This review has limitations. First, more complete searches of usual databases (*e.g*., PubMed, CINAHL, Embase, and PsycInfo) on the different topics of the area (screening for cancer, risk of developing the disease, mortality, intervention) are necessary. Second, we conducted a narrative review that had the aim to report the main data and results from significant studies in the area of the relationship between SMI and cancer. Specific attention to the quality of the studies we presented was not paid, although all of them were also taken into consideration in the metanalyses we cited.

## CONCLUSION

In conclusion, it is extremely important to create core-curricula for both professionals working in the oncology, primary or palliative care and professionals working in mental health and psychiatry settings [83], and to increase access to all possibilities of care for people with SMI. This would facilitate better physical clinical care for a fragile and extremely vulnerable segment of the population such as people with SMI [79]. A series of integrated multidisciplinary programs of screening and management for patients with SMI have been successfully developed [84-86], and these examples should be followed by other centers and gradually routinely implemented.
